# Preclinical evaluation of CD70-specific CAR T cells targeting acute myeloid leukemia

**DOI:** 10.3389/fimmu.2023.1093750

**Published:** 2023-02-10

**Authors:** Gongqiang Wu, Shanshan Guo, Qian Luo, Xiaoxia Wang, Wenhai Deng, Guifang Ouyang, Jeffrey J. Pu, Wen Lei, Wenbin Qian

**Affiliations:** ^1^ Department of Hematology, Dongyang Hospital Affiliated to Wenzhou Medical University, Dongyang People’s Hospital, Dongyang, Zhejiang, China; ^2^ Department of Hematology, The Second Affiliated Hospital, College of Medicine, Zhejiang University, Hangzhou, China; ^3^ Key Laboratory of Laboratory Medicine, Ministry of Education, School of Laboratory Medicine and Life Sciences, Wenzhou Medical University, Wenzhou, Zhejiang, China; ^4^ Hematology Department of Ningbo First Hospital, Ningbo Clinical Research Center for Hematologic Malignancies, Ningbo, China; ^5^ Department of Medicine, University of Arizona National Cancer Institute (NCI) Designated Comprehensive Cancer Center, Tucson, AZ, United States; ^6^ Research Center for Life Science and Human Health, Binjiang Institute of Zhejiang University, Hangzhou, China

**Keywords:** CD70, CAR-T, immunotherapy, leukemia stem cells, acute myeloid leukemia

## Abstract

**Backgrounds:**

Chimeric antigen receptor (CAR)-T cell therapy has achieved unprecedented success in treating hematopoietic malignancies. However, this cell therapy is hampered in treating acute myeloid leukemia (AML) due to lack of ideal cell surface targets that only express on AML blasts and leukemia stem cells (LSCs) but not on normal hematopoietic stem cells (HSCs).

**Methods:**

We detected the CD70 expression on the surfaces of AML cell lines, primary AML cells, HSC, and peripheral blood cells and generated a second-generation CD70-specific CAR-T cells using a construct containing a humanized 41D12-based scFv and a 41BB-CD3ζ intracellular signaling domain. Cytotoxicity, cytokine release, and proliferation in antigen stimulation, CD107a assay, and CFSE assays were used to demonstrate the potent anti-leukemia activity in vitro. A Molm-13 xenograft mouse model was established to evaluate the anti-leukemic activity of CD70 CAR-T *in vivo*. CFU assay was explored to assess the safety of CD70 CAR-T on HSC.

**Results:**

CD70 heterogeneously expressed on AML primary cells, including leukemia blasts, leukemic progenitor, and stem cells, but not expressed on normal HSCs and majority of blood cells. Anti-CD70 CAR-T cells exhibited potent cytotoxicity, cytokines production, and proliferation when incubated with CD70^+^ AML cell lines. It also displayed robust anti-leukemia activity and prolonged survival in Molm-13 xenograft mouse model. However, such CAR-T cell therapy did not completely eliminate leukemia *in vivo*.

**Discussion:**

Our study reveals that anti-CD70 CAR-T cells are a new potential treatment for AML. However, such CAR-T cell therapy did not completely eliminate leukemia *in vivo*, suggesting that future studies aiming to generate innovative combinatorial CAR constructs or to increase CD70 expression density on leukemia cell surface to prolong the life-span of CAR-T cells in the circulation will be needed in order to optimize CAR-T cell responses for AML.

## Introduction

Acute myeloid leukemia (AML) is the most common acute leukemia among adults. The incidence of AML increases with age, with a median age at diagnosis around 70 years old (1, 2). Currently, intensive chemotherapy and allogeneic hematopoietic stem cell transplantation (allo-HSCT) are the standard treatments for AML. However, the elderly patients and the patients with severe comorbidity usually couldn’t tolerate these harsh treatments. Besides, most patients who achieved complete remission after intensive chemotherapy will relapse even after receiving allo-HSCT (3, 4). With the advance of next-generation sequencing, we have gained more understanding of the molecular pathogenesis of AML, which leads to the application of several targeted therapies to inhibit crucial mutations and overexpression of oncogene for leukemia progression, such as FLT3, IDH1/IDH2, and BCL2 (5–8). However, the overall prognosis for AML patients is still poor with a 5-year survival of 30.5%, demonstrating an unmet need for innovative treatment strategies (9).

Chimeric antigen receptor (CAR)-T immunotherapy has reached remarkable success in treating multiple myeloma and B-cell malignancies and is entering a rapid development period globally (10–12). By now, much attention has been caught up on applying CAR-T immunotherapy to AML patients, although research has been impeded by the absence of exclusive cell surface target antigen. An ideal AML-specific antigen should be widely expressed on the surface of AML blasts and leukemic stem cells (LSCs) but rarely on normal tissues and hematopoietic stem cells (HSCs) to avoid off-target toxicities, especially the myelosuppressive effects. More than 20 AML cell surface antigen targets, such as CD33, CD123, CLL-1, FLT3, NKG2D, CD38, and CD7, have been evaluated in preclinical models and clinical trials (13). However, the drawback of such targets would be the limitation of on-target and off-tumor toxicities, which may not be tolerable. For example, CD33 and CD123 are often expressed on normal HSCs, and CAR-T cell targeting may impair subsequent hematopoiesis (14). In addition, CLL-1 is highly expressed in normal granulocytes, and severe pancytopenia was observed in all AML patients who received CAR-T cell therapy targeting CLL-1 in a first-in-human phase I study (15).

CD70 is a member of the tumor factor superfamily and plays an important role in immune responses through interaction with CD27 (16). It has been found to be highly expressed on the surface of several solid and hematological tumors, correlating with inferior prognosis in breast cancer, renal cell carcinoma, and some lymphomas (17). In AML, CD70 expression was demonstrated not only in the majority of patient samples but also in LSCs. Importantly, CD70 is absent on normal HSCs and exists transiently in some immune cells, making it a promising therapeutic target for AML patients (18, 19). Recent studies showed that targeting CD70 with a monoclonal antibody (mAb) or CAR-T cells could elicit strong anticancer activity against AML and B cell malignancies (20–22). Moreover, experiments demonstrated that CD70 mAb combined with hypomethylating agent, which can upregulate the expression of CD70 on leukemic cells, eliminated LSCs both *in vitro* and *in vivo (* 20). More recently, Sauer and colleagues (23) showed that CD70-specific CAR-T cells that contain either a single-chain variable fragment (scFv) or human CD27, the ligand of CD70, have potent activity against AML cells without impairing hematopoiesis.

We previously developed a second-generation CD70-specific CAR-T cells using a vector that contained a humanized 41D12-based scFv and 41BB-CD3ζ intracellular signaling domain (22). To determine the feasibility of treating AML patients with the CD70-CAR-T cells, we compared CD70 expression between AML cells, including cell lines and primary leukemic cells obtained from AML patients and human normal blood cells and HSCs. The results revealed substantial heterogeneity of CD70 expression among AML cells. Furthermore, we evaluated the potential anti-leukemia effects of this CD70-CAR-T cell construct.

## Methods

### Ethical statement

The animal studies were approved by the Institutional Animal Care and Use Committee of the Second Affiliated Hospital, College of Medicine, Zhejiang University. All samples from patients used in this study, which included peripheral blood (PB) and bone marrow (BM) samples, were obtained after receiving informed consent. The study was approved by the Ethics Committee of the Second Affiliated Hospital, College of Medicine, Zhejiang University.

### Cell culture

The following cell lines were purchased from American Type Culture Collection (ATCC, Manassas, VA): HEK293T/17(RRID: CVCL_1926), K562(RRID: CVCL_UC15), HL-60(RRID: CVCL_0002), Kasumi-1(RRID: CVCL_0589), U937(RRID: CVCL_0007), KG-1α(RRID: CVCL_1824) and THP-1(RRID: CVCL_0006). MOLM-13(RRID: CVCL_2119) was purchased from Beyotime Biotechnology (Shanghai, China) and identified by short tandem repeat (STR). All cell lines were mycoplasma-free tested by MycoAlter (Lonza Bioscience, USA). K562(RRID: CVCL_UC15), HL-60(RRID: CVCL_0002), Kasumi-1(RRID: CVCL_0589), U937(RRID: CVCL_0007), KG-1α(RRID: CVCL_1824), THP-1(RRID: CVCL_0006), and MOLM-13(RRID: CVCL_2119) were cultured in RPMI 1640 (Sigma, Germany) with 10% fetal bovine serum (FBS; Gibco, USA) and 1% penicillin/streptomycin (Gibco, USA).HEK293T/17(RRID: CVCL_1926) was cultured in DMEM (Gibco, USA) supplemented with 10% FBS, 1% penicillin/streptomycin (Gibco, USA), and 1% sodium pyruvate (Gibco). Normal HSCs were collected from the BM of healthy donors using CD34-specific microbeads (cat#130-046-702, Miltenyi Biotec, Germany) and cultured in IMDM (Sigma, Germany) with 20% FBS (Gibco, USA) for a short time.

### Generation of CD70-specific CAR T cells

The CD70-CAR T cells were generated as our previous publication (22). Briefly, CD70 and CD19 CAR contain a humanized 41D12-based scFv or FMC63 mAb, respectively, followed by the CD8α-based extracellular spacer and transmembrane domains with 41BB-CD3ζ intracellular signaling domain. CD3^+^ T cells were purified from peripheral blood mononuclear cells (PBMNCs) of healthy donors and were activated with CD3/CD28 dynabeads (Gibco, USA), which were then transfected with lentivirus encoding CD70 or CD19 CAR with 10 μg/mL polybrene (Sigma, Germany) at a multiplicity of infection of 20 (Sigma, Germany). The CD3/CD28 dynabeads were removed on days 3-5, and the transfected T cells were expanded in AIM-V (Gibco, USA) medium supplemented with 10% FBS (Gibco, Australia), 300 IU/mL IL-2, 5 ng/mL IL-7 and IL-15 for approximately 10 days.

### Flow cytometry

Flow cytometry was performed using a NovoCyte cytometer (ACEA, Agilent, Santa Clara, CA, USA). The following antibodies were purchased from Biolegend (San Diego, CA, USA): CD3-FITC (clone OKT3, RRID: AB_571907), CD3-APC(clone OKT3, RRID: AB_1937212), CD107a-PE-Cy7(clone H4A3, RRID: AB_11147955), CD70-PE-Cy7 (clone 113-16, RRID: AB_2687254), PE anti-Streptavidin (clone 3A20.2, RRID: AB_2571915), CD34-PE (clone 581, RRID: AB_1731862), CD33-FITC (clone HIM3-4, RRID: AB_314344), CD123-APC (clone 6H6, RRID: AB_439779), CD13-PerCP/Cyanine5.5 (clone WM15, RRID: AB_2561921), CD117-PE/Dazzle™594 (clone 2B8, RRID: AB_2564055), CD38-Alexa Fluor^®^700 (clone HB-7, RRID: AB_2566424), CD45-Pacific blue (clone HI-30, RRID: AB_493655). Biotinlyted CD70 protein (cat#CDL-H82D7, AcroBiosystems) was purchased from AcroBiosystems (Beijing, China), anti-mouse FMC63 (clone R19M, RRID: AB_2857947, BioSwan Laboratories) antibody was purchased from BioSwan Laboratories (Shanghai, China).

### CD107a degranulation assay

T cells and tumor cells were co-cultured (E:T = 1:1) in AIM-V medium, supplemented with 10% FBS and cytokines that included 300 IU/mL IL-2, 5 ng/mL IL-7, and 5 ng/mL IL-15 for 4 h at 37˚C. The GolgiStop protein transport inhibitor (cat#554724, BD Bioscience) and CD107a PE-cy7 antibody (clone H4A3, RRID: AB_11147955) were added in the culture medium. The cells were then labeled with Biotinlyted CD70 protein (cat# CDL-H82D7, AcroBiosystems) for 40 min, followed by 20 min with PE anti-Streptavidin (clone 3A20.2, RRID: AB_2571915) and CD3-APC (clone OKT3, RRID: AB_1937212), and then were analyzed by flow cytometry analysis. The percentage of CD107a^+^ cells was gated on live CD3^+^ CD70CAR^+^ cells for anti-CD70 CAR-T cells and on live CD3^+^ cells for control T (CT) cells.

### CAR-T cells proliferation assay

The control T cells and anti-CD70 CAR-T cells were labeled with CellTrace™ carboxyfluorescein diacetate succinimidylester (CFSE) Cell Proliferation Kit (Thermo Fisher) according to instructions and co-cultured with mytomycin-treated tumor cells for the specific monitoring time at an E:T ratio of 1:2. Then flow cytometry was used to detect T cell growth. All flow cytometry data were gated and analyzed using FlowJo software (version 10.7.1, RRID: SCR_008520).

### Cytotoxicity assays

The experimental group (X): Luciferase-expressing tumor cell lines (1.0 × 10^4^) were co-cultured with CT or CAR-T cells at various E:T ratios in a 96-well plate. The maximal cell killing group (Max) was treated with Triton X-100, while no T cells were added for the minimal cell killing group (Min). The cell suspension was harvested after 4 h and then centrifuged at 1500 rpm for 5 min. The supernatant was carefully discarded, and cells were resuspended with 100 μL 1.5 mg/mL of D-Luciferin Salt Bioluminescent substrate (PerkinElmer), and measured by a Specctramax i3 instrument (Molecular Devices, Sunnyvale, USA). The specific lysis was calculated using the following formulas: specific lysis = (Min − X)/(Min − Max) × 100%. Each experiment was repeated at least 3 times.

### Long-term cytotoxicity

Firefly luciferase-GFP expressing tumor cell lines were co-cultured with CT cells or CAR-T cells at E:T ratios of 1:1 or 1:5 in complete AIM-V medium supplemented with 10% FBS (Gibco), 300 IU/mL IL-2, 5 ng/mL IL-7 and 5 ng/mL IL-15. Cells suspension was mixed and detected at the indicated time after being labeled with CD3-APC (clone OKT3, RRID: AB_1937212) and 7-AAD (Biogems). The percentage of tumor (GFP^+^) and CT/CAR-T cells (CD3^+^) were then gated on 7-AAD negative cells. The co-culture system is replenished with fresh medium every 2-3 days.

### Cytokine release assay

Tumor cell lines were co-cultured with CT or CAR-T cells for 4 h at the indicated E:T ratios. Then the supernatant was harvested to measure released cytokines using Cytometric Bead Array (CBA) Human Th1/Th2 Cytokine Kit (BD, Franklin Lakes, NJ, USA). The data were analyzed using BD Biosciences CBA analysis software according to the instructions.

### Colony formation assay

The CD34^+^ cells were obtained from the healthy donors, and magnetic-bead-labeled antibodies were used to purify HSCs. Then HSCs were co-cultured with control T or CAR-T cells at an E:T ratio of 1:10 for 4 h. Untreated CD34^+^ cells were used as a negative control. The cell suspension was then labeled with CD34-PE (clone 581, RRID: AB_1731862) and CD3-APC (clone OKT3, RRID: AB_1937212) antibodies followed by flow cytometry analysis or plated in MethoCult™ (cat#H4034, Stemcell) for colony formation assay. Burst-forming unit-erythroid (BFU-E), colony-forming unit-granulocyte, macrophage (CFU-GM), and colony-forming unit-granulocyte, erythroid, macrophage (CFU-GEMM) counts were performed under a microscope after the incubation period of 14 days.

### *In vivo* studies

Molm-13 (RRID: CVCL_2119) cells (1.0 × 10^5^) expressing firefly luciferase-GFP were intravenously injected into 6-8 weeks old NOD.Cg-Prkdc^scid^ IL2rgtm^1Wjl^/SzJ (NSG)(RRID: IMSR_JAX:005557) mice (Biocytogen, Beijing, China). All mice used in this study were female. After 3 days, mice were blindly randomized to three groups (N = 8) and given either CT or CAR-T cells (2.0 × 10^6^) intravenously on day 0. Mice were euthanized by carbon dioxide inhalation, and then tumor growth was monitored using an IVIS lumina II *in vivo* imaging system (Caliper Biosciences, now PerkinElmer, Waltham, MA, USA). No subjects were excluded from our study. Responses were then scored by an experimenter blinded to injection condition and experimental cohort. The collected venous blood from the orbital vein on day 10 were used for complete blood count (CBC) and biochemistry tests. The heart, liver, spleen, lung and BM were obtained on day 18 after euthanized for bioluminescence imaging (BLI) and cytotoxicity assay was performed as described above.

### Statistical analysis

All data were performed with GraphPad Prism (version 9.0, RRID: SCR_002798) and presented as mean ± standard deviation (M ± SD). Student’s t-test, Turkey’s test, or one-way ANOVA was used. Log-rank analysis was used for mice survival data. All tests were two-tailed. The signs indicating statistical signification are as follows: *P* < 0.05 (*), *P* < 0.01 (**), *P* < 0.001 (***), *P* < 0.0001 (****), and *P* ≥ 0.05 (ns). The sample size was based on estimations by power analysis with a level of significance of 0.05 and a power of 0.9.

## Results

### CD70 is abundant in primary AML blast and LSCs but absent on majority of normal blood cells and HSCs

**Figure 1:**
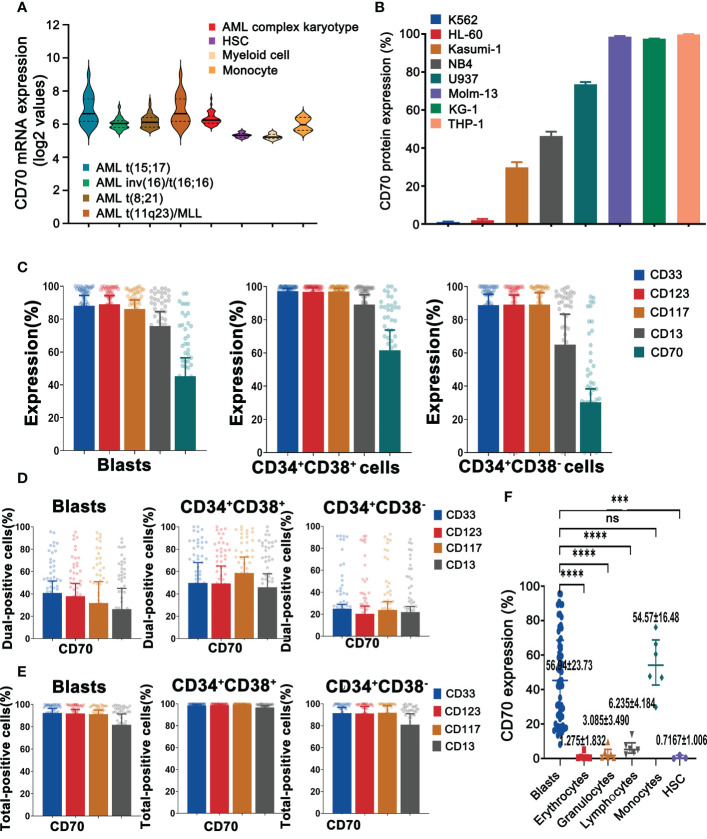
AML displays remarkable heterogeneity in CD70 expression. **(A)**, The mRNA expression levels of CD70 in human AML cells versus normal HSCs, myeloid cells, and monocytes by meta-analysis of BloodSpot databases (www.bloodspot.eu). Data are mean ± s.d. (AML, *n* = 241; HSCs, *n* = 6; myeloid cells, *n* = 8; monocytes, *n* = 4). **(B)**, CD70 protein expression of AML lines evaluated by flow cytometry. K562 cell line, a chronic myeloid leukemia cell line, was used as a negative control. **(C)**, Expression of CD70, CD33, CD123, CD117, and CD13 in AML blasts, leukemic progenitor cells and LSCs, which were obtained from AML patients (*n*=62) was analyzed by flow cytometry. **(D)**, Co-expression of CD70 and CD33, CD123, CD117, or CD13 in AML blasts, progenitor cells and LSCs, respectively, which assessed by flow cytometry. **(E)**, Additive expression of above antigen pairs in AML blasts, progenitor cells and LSCs was analyzed by flow cytometry. **(F)**, CD70 expression in HSCs and human blood cells including erythrocytes, granulocytes, lymphocytes, and monocytes compared with AML blasts. Tukey’s test was used for *P* values, ***P<0.001, ****P<0.0001, ns means no significance.

Firstly, we analyzed CD70 mRNA expression in primary AML cells, normal blood cells, and HSCs. Meta-analysis of Bloodspot databases (GSE13159 and GSE42519) indicated that CD70 mRNA is overexpressed in primary leukemic cells and LSCs obtained from different subtypes of AML patients in comparison to normal myeloid cells and HSCs. It is also upregulated in normal monocytes (**Figure 1A**). Consistent with this result, the comprehensive analysis of mRNA expression profiles of CD70 obtained from the Broad Institute Cancer Cell Line Encyclopedia (CCLE) also revealed that CD70 is highly expressed in several solid and hematologic malignancies including AML (**Supplementary Figure 1**). Thus, we next evaluated CD70 protein expression on different AML cell lines and primary leukemic cells that obtained from BM samples of 62 AML patients. Clinical characteristics are summarized in **Supplementary Table 1**. The K562 cells, a chronic myeloid leukemia cell line, and healthy human BM and PBMNCs were used as control. CD70 positivity (≥10%) was observed in the majority of AML cell lines with varying degrees of positive rates and median fluorescence intensity (MFI). The frequency of CD70^+^ cells in Kasumi-1, NB4, U937, KG-1α, Molm-13, and THP-1 was 29.83%, 46.37%, 73.52%, 97.54%, 98.59%, and 99.78%, respectively. Interestingly, HL-60 cells didn’t express CD70 (**Figure 1B**). The above data indicate that CD70 heterogeneously expresses among AML cell lines.

Furthermore, the patient BM samples (n=62), healthy human BM and PBMNCs were analyzed for CD33, CD123, CD117, CD13, and CD70 protein expression using flow cytometry. As expected, CD70 was differentially expressed on AML blasts (CD45^dim^SSC^lo^), leukemic progenitor cells (CD45^dim^SSC^lo^CD34^+^CD38^+^), and LSCs (CD45^dim^SSC^lo^CD34^+^CD38^−^) (**Figure 1C** and **Supplementary Figure 2A**). The frequency of CD70^+^ cells in these three kinds of primary cells was 45.25% (23.95%-68.59%), 61.57% (28.59%-86.43%), and 30.25% (12.71%-50.50%) respectively. Interestingly, CD33 and CD123, used as targets in CAR-T cell therapy for AML (13) were highly expressed in all primary leukemic cells.

To address the challenge of clonal heterogeneity, the bispecific CAR-T cells with a broadened range of tumor antigen recognition have been developed (24, 25). We therefore analyzed the combinatorial expression of CD33, CD123, and CD70 in primary AML cells. Forty-one percent of AML blasts expressed CD70 and CD33 while 38% of blasts had co-expression of CD70 and CD123. Similar results were also observed in LSCs (**Figure 1D**). However, total positivity for these pairs was significantly higher than dual-positivity, reaching 92.3% and 91.8% FACS positivity in blasts, 91.5% and 91.3% in LSCs, respectively (**Figure 1E**). Finally, CD70 was also found to be expressed in normal monocytes, with the mean expression level of 54.57%, and in lymphocytes (6.24%), but to be absent in erythrocytes, granulocytes, and HSCs (**Figure 1F** and **Supplementary Figure 2B**).

### Anti-CD70 CAR-T cells exhibited potent anti-tumor activity *in vitro*

**Figure 2:**
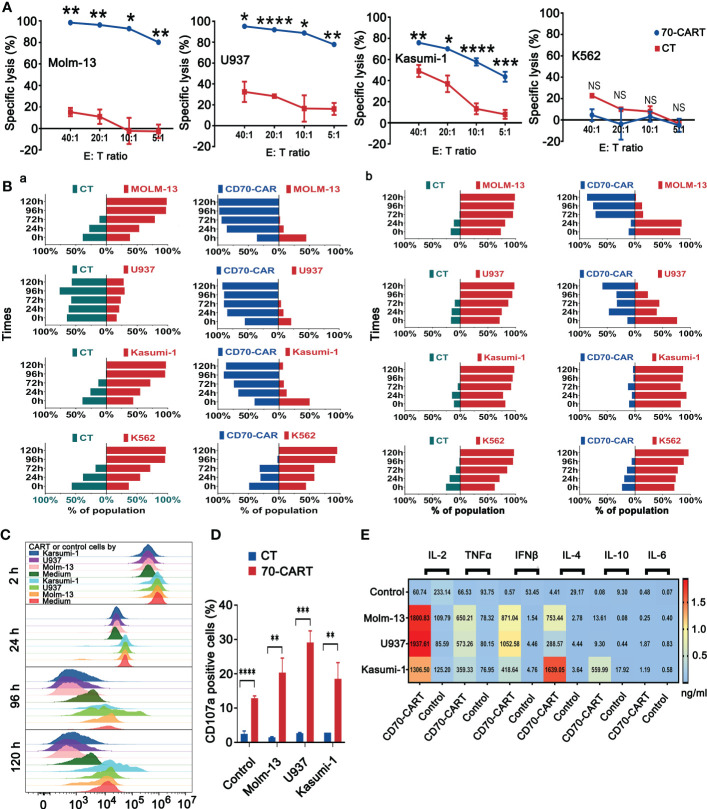
CD70 CAR-T cells exhibited potent cytotoxicity against CD70^+^ AML cell lines. **(A)**, Killing of AML cell lines by CD70-CAR-T cells as determined by luciferase-based cytotoxicity assay after 4 h co-culture at the indicated E:T ratios. K562 cell line was used as control. Data are mean ± s.d. from three independent experiments. Tukey’s test was used for *P* values, *P<0.05, **P<0.01, ***P<0.001, ****P<0.0001, ns means no significance **(B)**, Three kinds of AML cell lines and K562 cells expressing GFP were co-cultured with CD70-CAR-T cells or control T cells at the E:T ratio of 1:1 **(a)** and 1:5 **(b)**, respectively. At the indicated times, cells were harvested from co-culture system and then analyzed for CD3^+^ T cells, CAR^+^ T cells and GFP^+^ leukemic cells. Bars indicated percentage of T cells (green), CAR-T cells (blue), and leukemic cells (red) in the co-cultured cells. **(C)**, The proliferation of CD70-CAR-T cells and control T cells was measured using CFSE assay after co-cultured with different AML cell lines at an E:T ratio of 1:2 for the indicated times. **(D)**, AML cell lines were co-cultured with T cells or CD70-CAR-T cells for 5 h, respectively, and then the frequencies of CD107a-positive cells in each group were detected by flow cytometry. **P<0.01, ***P<0.001, ****P<0.0001. **(E)**, The supernatants obtained from co-cultures of AML cells and CAR T cells or control T cells were analyzed for cytokine production. The heatmap showing increased of cytokines including IL-2, TNF-α, IFN-γ, IL-4, and IL-10 in the groups of CAR-T cells.

We generated CD70-specific CAR-T cells by lentiviral transfection using CD70 scFv-based CAR construct (**Supplementary Figure 3A**). The culture process of CAR-T cells was depicted in **Supplementary Figure 3B**, and the efficiency of transfection was verified by flow cytometry (**Supplementary Figure 3C**), The Molm-13 (CD70^high^), U937 (CD70^int^), Kasumi-1 (CD70^low^) and K562 (CD70^neg^) cell lines expressing CD70 at different levels were used for *in vitro* study. We co-cultured anti-CD70 CAR-T and control T cells with these AML cell lines to explore their cytolytic capacity. Anti-CD70 CAR-T cells strongly induced cell lysis of Molm-13 (CD70^high^) and U937 (CD70^int^) cells, in a dose-dependent manner. We also observed that the CAR-T cells killed Kasumi-1 (CD70^low^) cells, albeit modestly (**Figure 2A**). Consistent with these results, anti-CD70 CAR-T cells were able to eliminate Molm-13 and U937 cells even at an E:T ratio of 1:5 when prolonged co-cultures were performed at *in vitro* study. Whereas the CAR-T cells displayed a relatively attenuated activity against Kasumi-1 cells and no cytotoxicity against K562 cells (**Figure 2B** and **Supplementary Figure 4**). Collectively, these data demonstrated that functional activity of anti-CD70 CAR-T cells largely correlated with CD70 expression on AML cells.

To better estimate the proliferation ability of CAR-T cells upon tumor challenge, we co-cultured anti-CD70 CAR-T and control T cells with three AML cells lines after being labeled with CFSE at an E:T ratio of 1:2. Anti-CD70 CAR-T cells showed strong proliferation ability in all tested time periods (**Figure 2C**). We also measured the CD107a degranulation and Th1/Th1 (IL-2, TNF-α, and IFN-γ) and Th2/Th2 (IL-4, IL-10, and IL-6) cytokines release of CAR-T cells after being co-cultured with AML cells lines. Anti-CD70 CAR-T cells demonstrated significantly increased CD107a degranulation and more Th1/Th1 cytokines and IL-4 release against AML cell lines (**Figures 2D, E**).

### Anti-CD70 CAR-T cells did not impair normal HSCs

**Figure 3:**
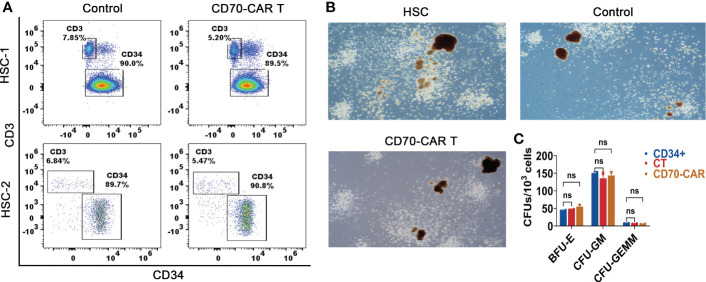
Anti-CD70 CAR-T cells did not impair normal HSCs. **(A)**, CD34^+^ cells were co-cultured with CAR-T or control T cells at an E:T ratio of 1:10 for 4 h, and then were analyzed for determining the frequencies of CD34^+^ and CD3^+^ cells. **(B)**, The cells obtained above co-culture system and methylcellulose were transferred into culture dishes for 14 days incubation. Representative images of colonies observed under the microscope **(C)**, The burst-forming unit-erythroid (BFU-E), colony-forming unit-granulocyte, macrophage (CFU-GM) and colony-forming unit-granulocyte, erythroid, macrophage (CFU-GEMM) counts were performed under a microscope. Data show the results of three independent experiment. One-way ANOVA analysis; ns means no significance.

Based on the aforementioned results and previous studies that demonstrated that CD70 is not expressed in normal HSCs, we next evaluated the cytotoxicity of anti-CD70 CAR-T cells to normal HSCs. As shown in **Figure 3A**, the number of CD34^+^ HSCs was unaffected by co-culture with anti-CD70 CAR-T cells. Moreover, the numbers of CFU-GM, BFU-E, and CFU-GEMM generated from human CD34^+^ cells, which were co-cultured with anti-CD70 CAR-T cells at an E:T ratio of 1:10 for 4 h, were almost the same as those generated from CD34^+^ cells cultured with control T cells or medium alone (**Figures 3B, C**). Taken together, our data suggest that anti-CD70 CAR-T cells preferentially kill AML cells but spare the HSCs.

### Anti-CD70 CAR-T cells were capable to reduce AML tumor burden and prolong survival time, but quickly attenuated in the circulation and resulted in leukemia progression in a xenograft model

**Figure 4:**
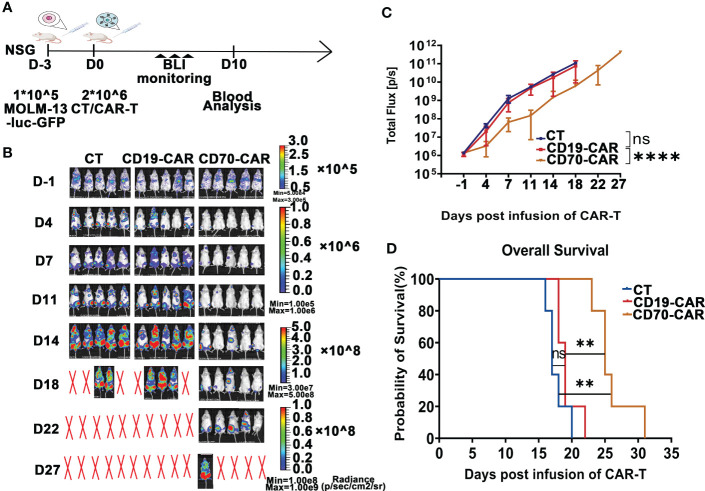
Anti-CD70 CAR-T cells exhibit a high capacity to reduce AML tumor burden and prolong survival time. **(A)**, Scheme of experimental design. On day 10, venous blood collected from orbital vein was used for complete blood count (CBC) and biochemistry tests. **(B)**, Molm-13 cells (1.0 × 10^5^) expressing luciferase-GFP were intraveously injected into NSG mice. Three days later, CAR-T or control T cells (2.0 × 10^6^) were given by intravenous injection. Bioluminescence imaging (BLI) was used to monitor leukemia burden at the indicated times. **(C)**, Leukemia burden (total flux) quantified in mice treated with control T cells, CD19-CAR-T, and CD70-CAR-T cells at the indicated days. One-way ANOVA, *post hoc* was Tukey’s test. ****P<0.0001; ns means no significance. **(D)**, Kaplan-Meyer plot showing mouse survival. *P* values from log-rank test. **P<0.01, ns means no significance.

We finally analyzed *in vivo* cytotoxic activity of anti-CD70 CAR-T cells against AML cells. Using Molm-13 cell line expressing a high level of CD70, which was stably expressing both GFP and luciferase, we established a NSG (NOD.Cg-Prkdc^scid^ IL2rg^tm1Wjl^/SzJ) xenograft model. After engraftment of the 1 × 10^5^ Molm-13 cells for 3 days, we injected a single dose of 2 × 10^6^ CAR T cells and monitored animals using *in vivo* imaging (**Figure 4A**). We found that in mice engrafted with AML cells, treatment with CD70-CAR-T cells resulted in a significant reduction in tumor burden and prolonged survival (**Figures 4B–D**); however, CD70-CAR-T cells became undetectable on day 18 post-infusion and eventually, all mice died from leukemia progression.

**Figure 5:**
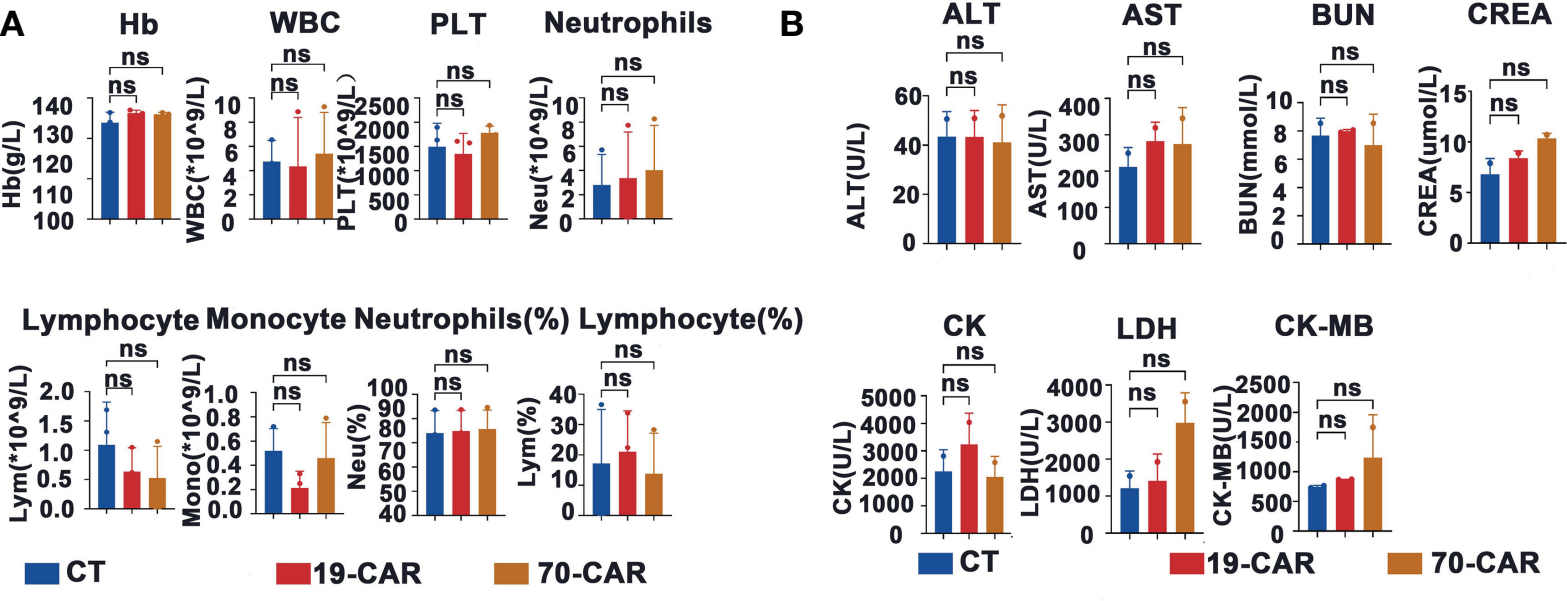
Evaluation of hematologic toxicity of CD70-CAR-T cells *in vivo*. **(A)**, Venous blood of the mice (n=3) treated with CAR-T or control T cells were collected on day 10. The data of hemoglobin (Hb), white blood cells (WBC), platelet (Plt), lymphocytes (Lym), neutrophils (Neu), and monocytes (Mono) were shown, **(B)**, The data of blood biochemistry including alanine aminotransferase (ALT), aspartate aminotransferase (AST), blood urea nitrogen (BUN), creatine kinase (CK), creatine kinase-MB (CK-MB), creatinine (CREA) and lactate dehydrogenase (LDH) were shown. One-way ANOVA analysis; ns means no significance.

We also examined potential red blood cell (RBC), white blood cell (WBC), and platelet toxicity upon administration of CAR-T cells. Post CAR-T cell infusion on day 10, blood cells were counted using an automated hematology analyzer in EDTA-anticoagulated whole blood samples (**Figure 5A**). Mice treated with CAR-T cells showed unchanged blood cell counts. Moreover, no alterations in serum chemistry parameters were seen in the mice receiving CAR-T cells (**Figure 5B**).

**Figure 6:**
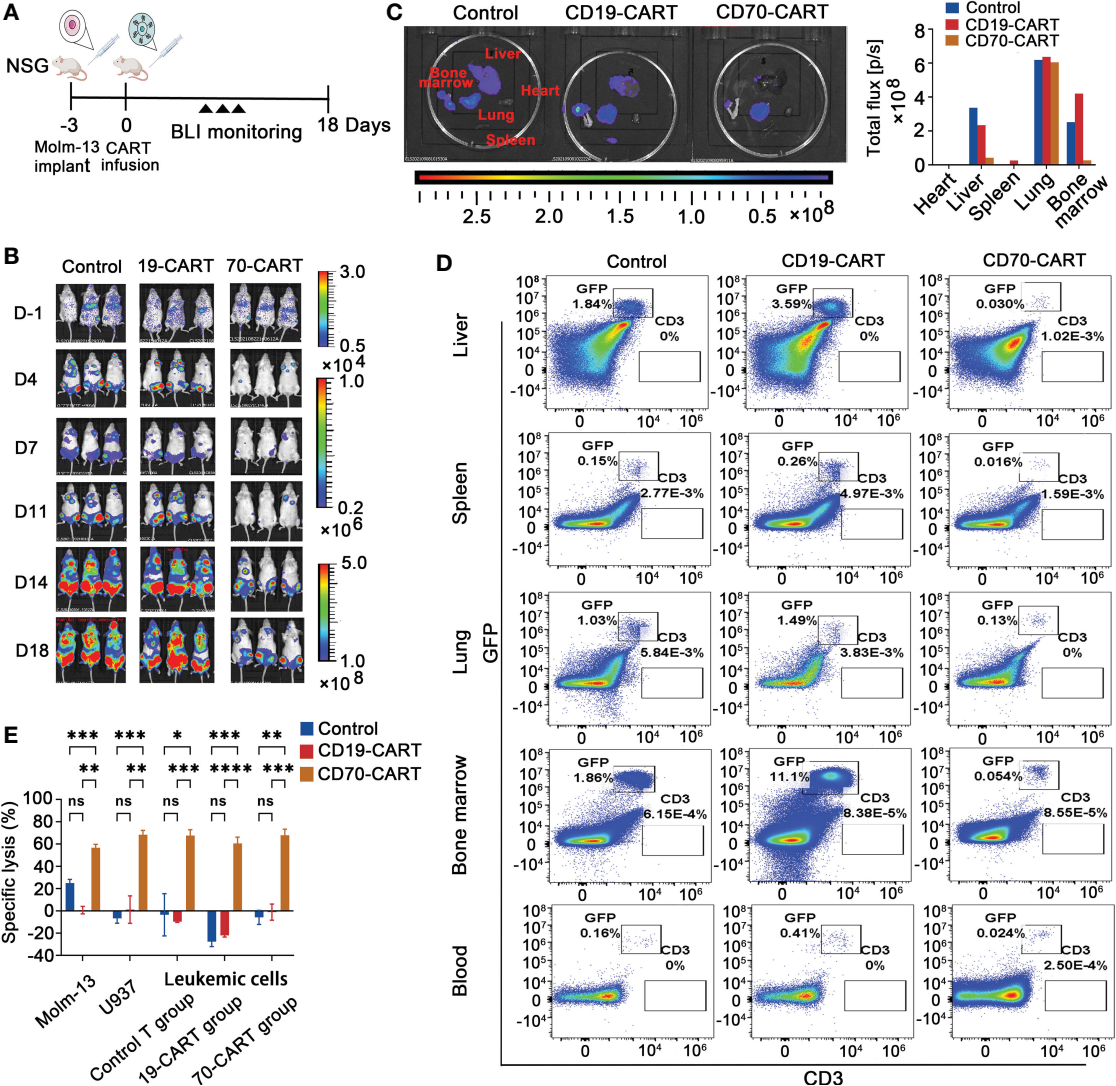
Evaluation of measurable residual disease (MRD) and CAR-T cell persistence *in vivo*. **(A)**, Scheme of *in vivo* experimental design. Leukemia bearing mice were also established using Molm-13 cells (1.0 × 10^5^), which were treated with CAR T cells. Control T cells were used as control. **(B)**, Tumor burden was monitored using BLI. **(C)**, On day 18, mice (n=3) were killed, and heart, liver, spleen, lung and bone marrow (BM) were taken and followed with BLI to measure tumor burden, and then total flux in these organs were quantified. **(D)**, Suspension cells extracted from above organ except heart and peripheral blood of mice were analyzed for leukemia cells and CD3 positive T cells by flow cytometry. Representative results were shown. **(E)**, The leukemic cells, obtained from BM of mice that were treated CAR-T or control T cells, were cultured for 3 days, and then were co-cultured with control T, and CAR-T cells at E:T ratio of 10:1 for 4 h. Molm-13 and U937 cell lines were used as control. Killing of leukemic cells by CD70-CAR-T cells as determined by luciferase-based cytotoxicity assay. One-way ANOVA, *post hoc* using Tukey’s test. *P<0.05, **P<0.01, ***P<0.001, ****P<0.0001, ns means no significance.

To investigate potential resistance mechanisms to CD70-CAR-T cell therapy *in vivo*, we performed another parallel animal experiment using Molm-13 xenograft mice (**Figure 6A**). Similarly, tumor inhibition has been observed after CD70-CAR-T cells infusion; however, mice bearing leukemic cells eventually relapsed (**Figure 6B**). We next analyzed the tumor burden of organs, including heart, liver, spleen, lung, and BM, which were obtained on day 18 after CAR-T cell infusion. BLI images showed that treatment with CD70-CAR-T cells significantly reduced tumor burden of liver, spleen, and BM compared with control treatments, but failed to eliminate leukemic cells in lung (**Figure 6C**). The cell suspension was then obtained from these organs and analyzed by flow cytometry for GFP^+^ leukemic cells and CD3^+^ T or CAR-T cells. As shown in **Figure 6D**, a high tumor burden was observed in the lung compared with other organs. Flow cytometry analysis also revealed that the leukemic cells that were separated from mouse BM were still CD70 positive (Supplementary **Figure 5**). These leukemic cells and AML cell lines U937 and Molm-13 cells were then cocultured with CAR-T cells, respectively. The cytotoxic activity of CD70-CAR-T cells was assessed. We found that CD70-CAR-T but not CT cells efficiently killed both leukemic cells obtained from mice and AML cell lines (**Figure 6E**).

## Discussion

The application of CAR-T cell therapy to patients with AML has been limited due to the lack of target antigens that can discriminate LSCs from normal HSCs (14). CD70 has recently received more attention as a potential target because it is both absent on normal HSCs and highly expressed on AML blasts and stem/progenitor cells (18–23). We previously generated two kinds of second-generation CD70-specific CAR-T cells using scFv derived from 41D12 mAb and truncated CD27 (trCD27). We demonstrated that scFv-based anti-CD70-CAR-T cells efficiently killed both CD19^+^ and CD19^-^ B-cell lymphoma cells (22). In this study, we investigated the function of CD70scFv-based CAR-T cells *in vitro* against a panel of AML cell lines that have different expression levels of CD70 for cytotoxicity, antigen-stimulated proliferation, and activation, and cytokine release. We found that CD70 is variably expressed by different AML lines and its expression level is largely correlated with the functional effectiveness of CD70-CAR-T cells, consistent with a previous report (26). Furthermore, relative to CD19-CAR-T cells and control T cells treatment, CD70-CAR-T cells significantly prolonged the survival of mice bearing AML cells and effectively inhibited tumor growth. Recently, Sauer et al (23). generated a panel of CD70-specific CAR-T cells and demonstrated that CD70-specific CAR-T cells transfected with CD27, the ligand of CD70, exhibited superior killing capacity compared with CD70scFv-based CAR-T cells *in vivo*. Moreover, a novel CD27-based CAR-T cells, in which CD8 hinge and transmembrane had been genetically modified, have improved immune synapse-binding avidity and increased expansion, eliciting more potent antileukemia activity *in vivo (* 26). Taken together, these studies demonstrate the feasibility of using anti-CD70-CAR T cells to effectively target AML that express CD70 antigen.

It was reported that CD70 expression varied in AML cell lines and primary cells, indicating the heterogeneity in both frequency and level of CD70-positive cell expression (19, 23). Here, we found that AML primary cells differentially expressed CD70. The frequency of CD70^+^ cells in AML blasts (CD45^dim^SSC^lo^), leukemic progenitor cells (CD34^+^CD38^+^) and LSCs (CD45^dim^SSC^lo^CD34^+^CD38^−^) was 45.25% (23.95%-68.59%), 61.57% (28.59%-86.43%), and 30.25% (12.71%-50.50%), respectively. Thus, broad utilization of CD70-specific CAR T cells in AML might be hindered by the heterogeneity of CD70 expression. Increasing CD70 expression density on leukemia cell surface can improve the efficacy of CD70-CAR-T cell-therapy. It was demonstrated that treatment with hypomethylating agents (HMA) led to upregulation of CD70 expressed on LSCs and targeting CD70-expressing LSCs by cusatuzumab, a human CD70 monoclonal antibody, resulted in elimination of LSCs *in vitro* and *in vivo (* 20). Importantly, the efficacy of combination therapy with cusatuzumab and HMA azacytidine was also validated in a phase 1/2 trial (20). In addition, many strategies have emerged in order to improve the capacity of CAR-T therapy, aiming to target multiple tumor antigens (19, 27–29). To address the challenge of CAR-T cell therapy in AML, He and colleagues (30) developed a combinatory bispecific and split CAR (BissCAR) T-cell system that targets CD13 and TIM3. In this system, anti-CD13 nanobody linked with CD3ζ recognized CD13 on both HSCs and LSCs, and anti-TIM3 scFv linked with CD28 and 4-1BB recognized TIM3 only on LSCs. Therefore, the Biss CAR-T cells could be activated only by LSCs but not by normal HSCs. Another strategy includes bispecific T cells that co-express two CARs or express a dual CAR, which recognize target cells that have any of two given antigens (19, 31). Given the fact that combinatorial antigenic targeting approaches are being explored for potential improved antitumor activity compared to conventional CAR T-cells, we further analyzed four combinatorial expression pattern: CD70 + CD33, CD70 + CD123, CD70 + CD117, and CD70 + CD13. We found that only about half of the AML primary cells coexpressing two given antigens, whereas cumulative expression of the paired antigens was approximately a hundred percent. This finding is consistent with antigenic heterogeneity of AML and provides a rationale to use these antigen pairs in the dual-targeting CAR-T cell therapy.

The mechanisms of resistance to CAR-T cell therapy in AML are poorly understood. Nonetheless, it is clear that antigen-negative relapse and antigen-positive relapse are the main mechanisms associated with resistance to CAR-T cell treatment. Antigen-negative relapses are related to CAR-driven mutations, alternative splicing, epitope masking, low antigen density, and lineage switching (32–34). Antigen-positive relapses are often due to poor CAR-T persistence, which may result from CAR immunogenicity, inherent T-cell quality, initial T-cell phenotype, co-stimulatory domain in each CAR construct, and the tumor microenvironment (TME) (35–38). In the present study, we observed that although promising, the mice bearing Molm-13 cells treated with CD70-CAR-T cells eventually showed disease progression, even though Molm-13 cells highly expressed CD70 while the CAR-T cells failed to maintain in circulation. Leukemia cells separated from BM of mice were still CD70 positive and sensitive to cell death mediated by CD70-CAR-T cells *in vitro*, indicating that antigen-positive relapse and limited persistence may contribute to the resistance to CD70-CAR-T treatment. Until recently, most CARs evaluated in preclinical or clinical have derived their single chain variable fragment (scFv) from the murine monoclonal antibody, such as FMC63. Mounting evidence suggests that this may generate a high level of anti-CAR antibodies response and cause the limiting persistence of the CAR-T (35). Thus, a fully human scFv-based CAR has been developed to overcome the issue of transgene immunogenicity (39). Furthermore, the limited persistence observed of the infused CAR-T cells could also be explained by the design of the CAR, which lacked a co-stimulatory domain, or the effect of the co-stimulatory domain. Several data suggest that CAR-T cells containing the CD28 domain persist up to 3 months, while those containing 4-1BB domain can persist up to 5 years (40, 41). The co-stimulatory domain used in CD70 CAR is also 4-1BB within this report. So other details of CAR design, such as specific characteristics of the antigen-binding domain, the presence and structure of an extracellular hinge region, and features of the transmembrane domain, might also affect the CD70-CAR-T cell attributes and wait for being optimized. Moreover, inherent T-cell quality or initial T-cell phenotype such as central memory T-cell (Tcm) and stem cell-like memory T-cells (Tscm) are also considered to be related to the proliferation and persistence of CAR-T cells (36). Thus, procedures inducing the transformation of CAR-T cells to Tcm and Tscm could be an alternative for preventing antigen-positive relapse by enhancing a durable response and persistence of the cells. In addition, the expression of T cell co-inhibitory receptors, including PD1, Tim-3, and LAG-3, was up-regulation on CAR-T cells resulting in possible inhibitory effects (42). Therefore, the combination therapy of immune checkpoint inhibitors and CAR-T cells represents another viable strategy for CAR-T cell expansion and persistence. Tumor heterogeneity and the role of the TME also hinder CAR-T expansion (38). Hypoxic tumor conditions, immunosuppressive immune cells such as regulatory T cells (Tregs), tumor-associated macrophages (TAMs), and myeloid-derived suppressor cells (MDSCs), immunosuppressive soluble factors, such as TGF-β, IL-4, and IDO, and expression of inhibitory receptors and ligands by tumor and/or stromal cells, such as PD1/PDL1, CTLA-4, LAG-3, could also impair CAR-T cell function during the CAR-T therapy *in vivo*. Therefore, overcome the resistance of TME could improve the effect of CAR-T. Understanding these obstacles to sustain the proliferation and persistence of the CAR-T cells may offer help to the generation of CAR-T cells with prolonged life-span *in vivo*.

In summary, this study provides evidence that CD70 is heterogeneously expressed on AML primary cells, including blasts, leukemic progenitor, and stem cells, but not expressed on normal HSCs and the majority of blood cells. The results also demonstrated potent anti-AML effects and the safety profile of CD70scFv-based CAR T cells both *in vitro* and *in vivo*. However, such CAR-T cell therapy did not completely eliminate leukemia in a xenograft mouse model. Future studies aiming to generate novel combinatorial CAR constructs, to increase CD70 expression density on leukemia cell surface, or to improve the persistence of CD70-CAR-T cells *in vivo* will be needed to optimize CAR T cell responses for AML.

## Data availability statement

The original contributions presented in the study are included in the article/**Supplementary Material**. Further inquiries can be directed to the corresponding authors.

## Ethics statement

The studies involving human participants were reviewed and approved by Ethics Committee of the Second Affiliated Hospital, College of Medicine, Zhejiang University. The patients/participants provided their written informed consent to participate in this study. The animal study was reviewed and approved by Institutional Animal Care and Use Committee of the Second Affiliated Hospital, College of Medicine, Zhejiang University. Written informed consent was obtained from the individual(s) for the publication of any potentially identifiable images or data included in this article.

## Author contributions

WQ, WL, and JP proposed the concepts. GW, SG, QL, XW, WD, and GO collected patient’s sample and performed the study. SG, QL, WQ and JP analyzed data and drafted the manuscript. All authors contributed to the article and approved the submitted version.

## Supplementary material

Supplementary Figure 1 mRNA expression profile of CD70. CD70 expression in various cancer cell lines based on Cancer Cell Line Encyclopedia (CLE) database.

Supplementary Figure 2 Flow cytometry gating strategy for detection of LSCs and HSCs. **(A),** Representative flow cytometry plots of the gating strategy used to determine CD70 protein expression on AML blasts, CD34^+^CD38^+^ progenitor and CD34^+^CD38^−^ LSCs from AML patients. **(B),** Flow cytometry plots of the gating strategy used to determine CD70 expression on erythrocytes, granulocytes, monocytes and lymphocytes from healthy donors.

Supplementary Figure 3 Transduction efficiency of CD70 CAR. **(A)**, Schematic representation of lentiviral constructs including anti-CD70 CAR and anti-CD19 CAR. B, The *in vitro* culture process of CAR-T cells. **(C)**, Flow cytometry plots showing the transduction efficiency of anti-CD70 CAR in human T cells used in the *in vivo* experiment.

Supplementary Figure 4 CD70-CAR-T cells exhibited potent cytotoxicity against CD70^+^ AML cell lines in a long term culture. Three kinds of AML cell lines including Molm-13, U937, Kasumi-1 cells and K562 cells were treated with anti-CD70 CAR-T cells at a E:T ratio of 1:1 **(A)** or 1:5 **(B)** for the indicated times. Then cells were analyzed for GFP^+^ and CD3^+^ cells.

Supplementary Figure 5 Leukemic cells obtained from mice that were treated with CAR-T cells showed CD70 positive. **(A)**, Flow cytometry gating strategy for detection of expression of CD70 and CD123 on leukemic cells. **(B)**, The leukemic cells, obtained from BM of mice that were treated CAR-T or control T cells, were analyzed using flow cytometry for detection of CD70 and CD123 expression.
