# Supplementary figures and images for: Preclinical evaluation of CD70-specific CAR T cells targeting acute myeloid leukemia
Source: Front Immunol. 2023 Feb 10;14:1093750. doi: 10.3389/fimmu.2023.1093750 (PMC9950117; doi:10.3389/fimmu.2023.1093750)

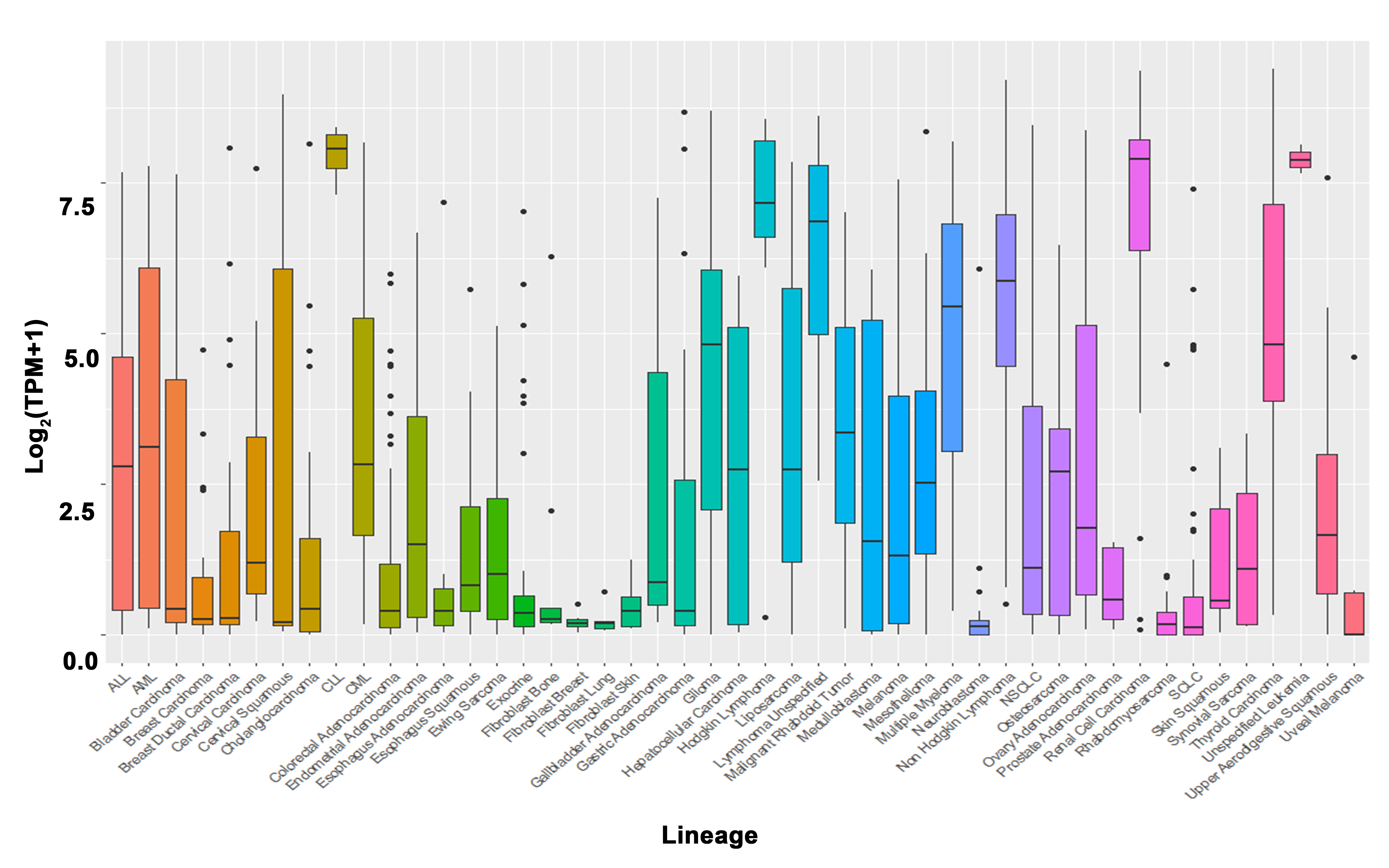

Supplement: Supplementary Figure 1 — mRNA expression profile of CD70. CD70 expression in various cancer cell lines based on Cancer Cell Line Encyclopedia (CLE) database. [file Image_1.jpeg]

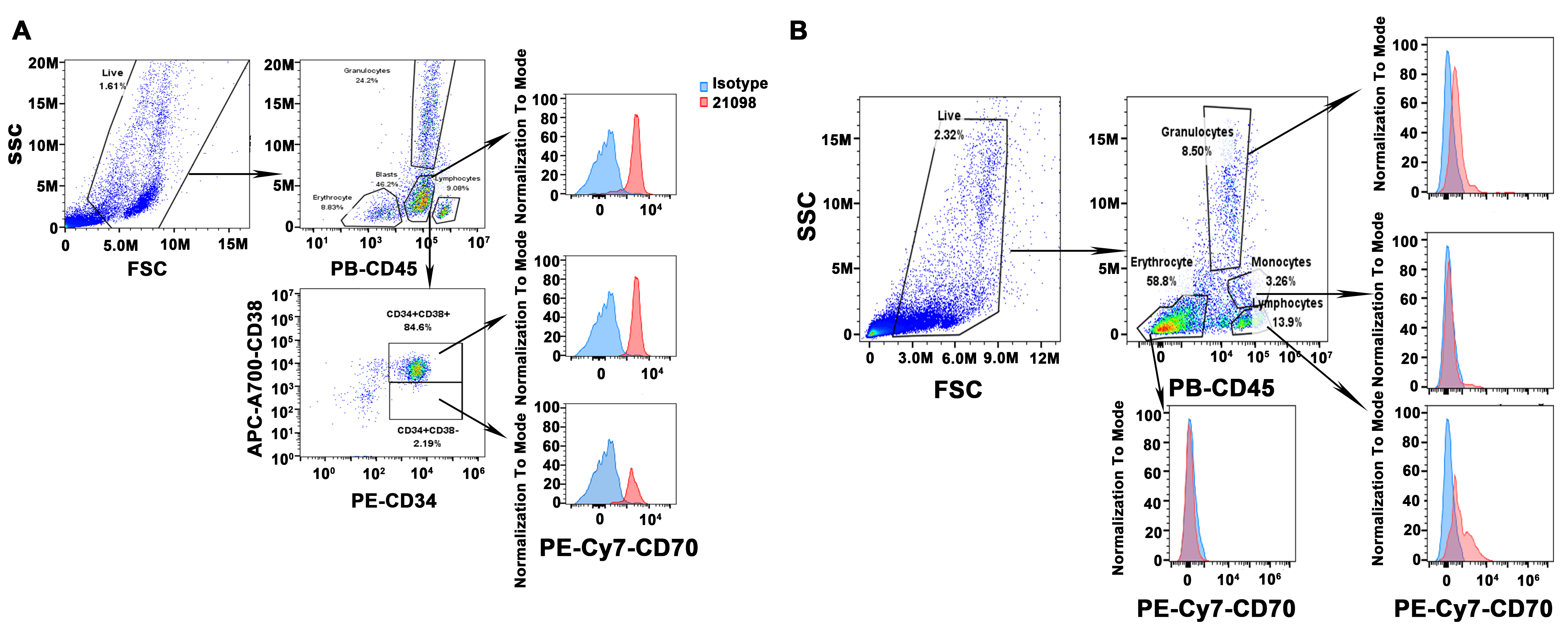

Supplement: Supplementary Figure 2 — Flow cytometry gating strategy for detection of LSCs and HSCs. (A), Representative flow cytometry plots of the gating strategy used to determine CD70 protein expression on AML blasts, CD34+CD38+ progenitor and CD34+CD38− LSCs from AML patients. (B), Flow cytometry plots of the gating strategy used to determine CD70 expression on erythrocytes, granulocytes, monocytes and lymphocytes from healthy donors. [file Image_2.jpeg]

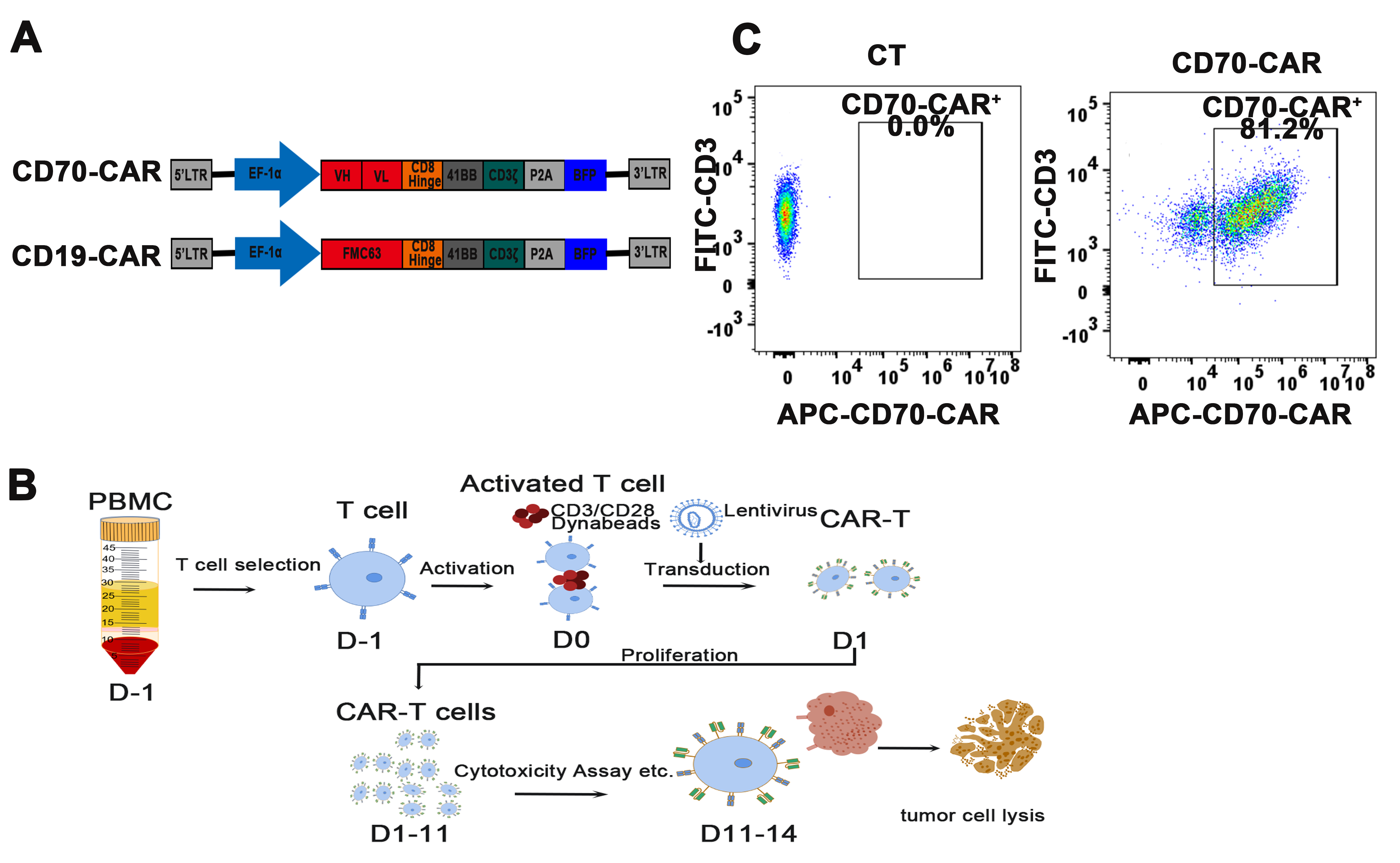

Supplement: Supplementary Figure 3 — Transduction efficiency of CD70 CAR. (A), Schematic representation of lentiviral constructs including anti-CD70 CAR and anti-CD19 CAR. B, The in vitro culture process of CAR-T cells. (C), Flow cytometry plots showing the transduction efficiency of anti-CD70 CAR in human T cells used in the in vivo experiment. [file Image_3.png]

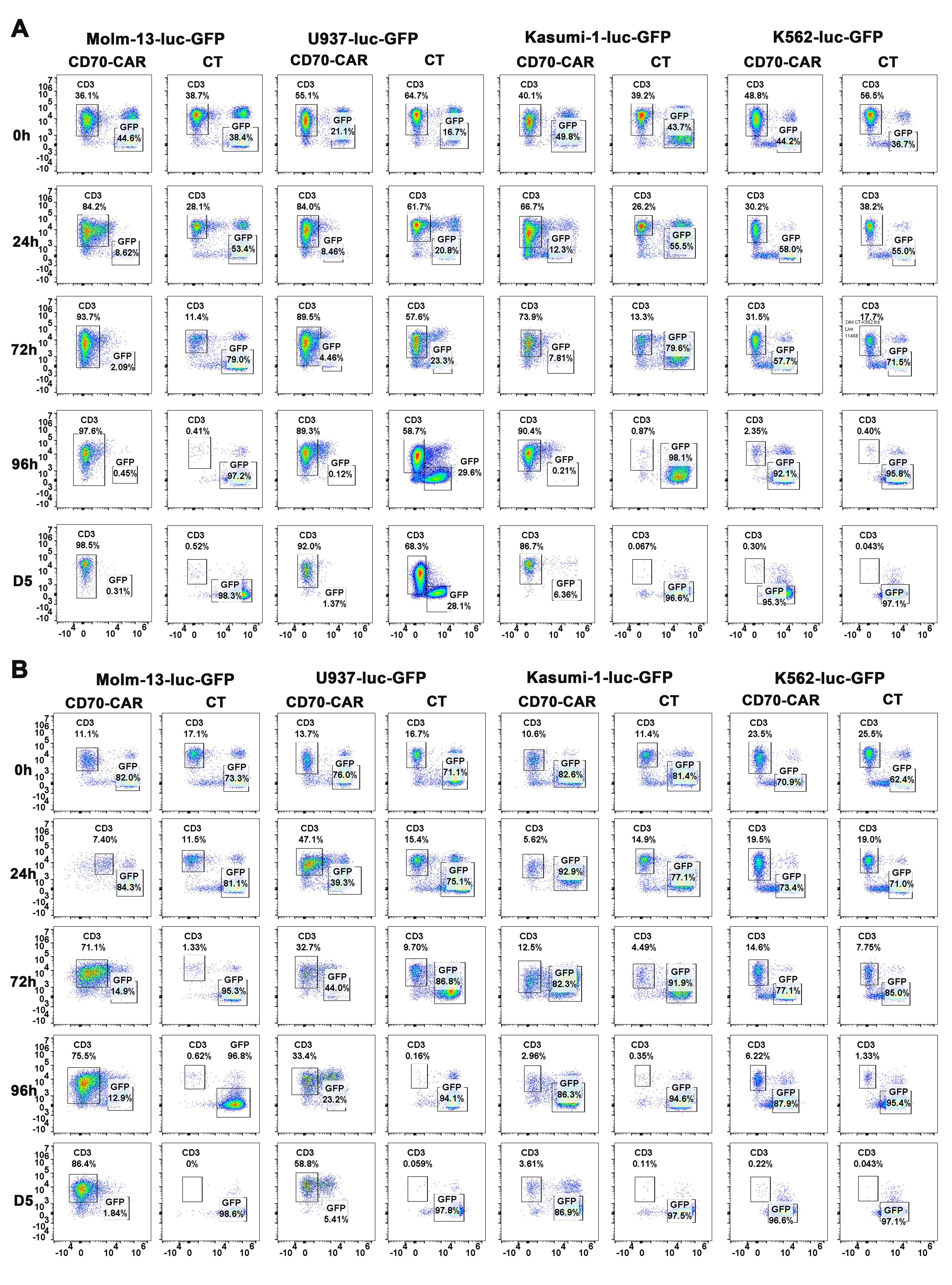

Supplement: Supplementary Figure 4 — CD70-CAR-T cells exhibited potent cytotoxicity against CD70+ AML cell lines in a long term culture. Three kinds of AML cell lines including Molm-13, U937, Kasumi-1 cells and K562 cells were treated with anti-CD70 CAR-T cells at a E:T ratio of 1:1 (A) or 1:5 (B) for the indicated times. Then cells were analyzed for GFP+ and CD3+ cells. [file Image_4.jpg]

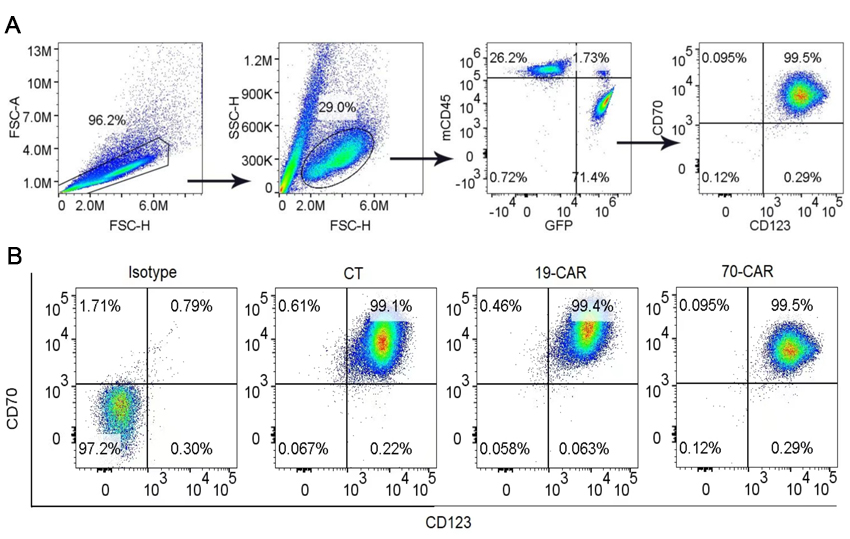

Supplement: Supplementary Figure 5 — Leukemic cells obtained from mice that were treated with CAR-T cells showed CD70 positive. (A), Flow cytometry gating strategy for detection of expression of CD70 and CD123 on leukemic cells. (B), The leukemic cells, obtained from BM of mice that were treated CAR-T or control T cells, were analyzed using flow cytometry for detection of CD70 and CD123 expression. [file Image_5.jpeg]
