# Supplementary material for: Preclinical evaluation of CD70-specific CAR T cells targeting acute myeloid leukemia
Source: Front Immunol. 2023 Feb 10;14:1093750. doi: 10.3389/fimmu.2023.1093750 (PMC9950117; doi:10.3389/fimmu.2023.1093750)
Supplement: Supplementary file 6 [file Table_1.docx]

**Supplementary Table 1.** The clinical characteristics of the patients and CD70 expression

|  | **Number** | **CD70（median(Quartiles)）** | ***P* value** |
| --- | --- | --- | --- |
| **Sex** |  |  | 0.7934 |
| Male | 36 | 47.86%（26.54%，64.57%） |  |
| female | 26 | 43.08%（21.73%，74.34%） |  |
| **Age** |  |  | 0.6410 |
| ＜60 | 38 | 43.32%（27.35%，68.59%） |  |
| ≥60 | 24 | 47.86%（18.75%，73.21%） |  |
| **FAB** |  |  | 0.5979 |
| M0 | 1 | 77.25% (77.25%,77.25%) |  |
| M1 | 5 | 61.24% (34.71%,76.72%) |  |
| M2 | 15 | 52.14% (25.12%,77.41%) |  |
| M4 | 13 | 45.13% (26.68%,76.18%) |  |
| M5 | 25 | 42.00% (17.04%,64.36%) |  |
| M6 | 1 | 75.68% (75.68%,75.68%) |  |
| M7 | 1 | 30.90% (30.90%,30.90%) |  |
| **Primary/Relapse** |  |  | 0.6162 |
| Primary | 55 | 45.13% (24.25%,64.98%) |  |
| Relapse | 7 | 59.43% (17.78%,77.41%) |  |
